# Performance and safety of therapeutic erythrocytapheresis in polycythemia and hemochromatosis treatment: single centre experience

**DOI:** 10.1016/j.htct.2024.05.011

**Published:** 2024-09-07

**Authors:** Iva Lucija Burnać, Ines Bojanić, Sanja Mazić, Marija Lukić, Branka Golubić Ćepulić

**Affiliations:** a Department of Transfusion Medicine, University Hospital Sveti Duh, Zagreb, Croatia; b Clinical Department of Transfusion Medicine and Transplantation Biology, University Hospital Centre Zagreb, Zagreb, Croatia; c University of Applied Health Sciences, Zagreb, Croatia; d School of Medicine, University of Zagreb, Zagreb, Croatia; e Department of Health Studies, University of Split, Split, Croatia

**Keywords:** Erythrocytapheresis, Therapeutic apheresis, Hemochromatosis, Polycythemia

## Abstract

**Introduction:**

Therapeutic erythrocytapheresis has some advantages over therapeutic phlebotomy, the standard treatment for cytoreduction in polycythemia and hemochromatosis. Erythrocytapheresis can be performed on different cell separators, each with its own characteristics. We present our experience of therapeutic erythrocytapheresis in the treatment of polycythemia and hemochromatosis with an analysis of the performance of cytoreduction, and a comparison between the characteristics of intermittent- and continuous-flow cell separators.

**Material and methods:**

During a 20-year period, 1731 procedures were performed in 125 patients, 1634 (94.4%) with a Haemonetics MCS+ separator and 97 (5.6%) with a Spectra Optia system device. The performance of cytoreduction using the Haemonetics MCS+ separator was analysed in 442 procedures performed in 56 patients and the performance of the two apheresis devices was compared.

**Results:**

Haemoglobin (Hb) and haematocrit (Hct) values were significantly reduced after erythrocytapheresis with the Haemonetics MCS+ device (Hb: 18.69%; Hct: 18.73%; p-values both <0.001). The reductions of Hb and Hct were significantly higher in the Haemonetics MCS+ procedure (p-value <0.001), but the Spectra Optia procedure depleted a significantly higher RBC volume (495 mL versus 442 mL) in a shorter time (18 min versus 36 min).

**Conclusion:**

Both the Haemonetics MCS+ and Spectra Optia systems proved to be highly efficient and safe in RBC cytoreduction with short procedure times. Erythrocytapheresis reduces the frequency of necessary procedures thereby justifying its therapeutic use especially in eligible patients of working age.

## Introduction

In conditions such as polycythemia and hemochromatosis, in which red blood cells or serum iron removal is the preferred therapeutic modality, treatment is traditionally performed by the withdrawal of whole blood using therapeutic phlebotomy.^1^, ^2^, ^3^, ^4^ When an apheresis device is available, in addition to therapeutic phlebotomy, the hematocrit (Hct) can be reduced by therapeutic erythrocytapheresis which enables the depletion of a larger volume of concentrated red blood cells (RBCs) from the patient's circulation, while plasma, platelets, and white blood cells remain stable.^5^ Automated RBC depletion can be performed with different types of centrifuged-based apheresis systems, each having its own advantages and disadvantages.^6^

The aim of this retrospective study was to present our experience with therapeutic erythrocytapheresis in the treatment of polycythemia (PV) and hemochromatosis (HH), analyse the performance of these systems for cytoreduction, and compare the characteristics of procedures using intermittent- and continuous-flow cell separators.

## Material and methods

### Patients

This single-centre retrospective study evaluated therapeutic erythrocytapheresis performed from 2001 to 2021 in the Clinical Department of Transfusion Medicine and Transplantation Biology, University Hospital Centre Zagreb. All procedures were performed for the treatment of PV or HH upon the request of the attending physician. All patients included in this study were previously treated with therapeutic phlebotomy and had adequate peripheral venous access for the stable blood flow required for apheresis procedures. Circulatory compensation mechanisms in maintaining normovolemia were assessed for each patient based on clinical examination and medical history with only hemodynamically stable patients being treated with erythrocytapheresis.

The Ethics Committee of the University Hospital Centre Zagreb approved this study.

### Erythrocytapheresis procedures

Procedures were carried out using two types of apheresis devices, the Haemonetics MCS+ (Haemonetics MCS+ Corporation, Braintree, MA, U.S.A.) and Spectra Optia Apheresis systems (Terumo BCT, Lakewood, CO, U.S.A.). Since 2001, erythrocytapheresis procedures have been performed on the intermittent-flow apheresis device, Haemonetics MCS+, using the TEA protocol that allows the use of a single vein for vascular access. Based on the patient´s sex, body weight, height and initial Hct, the system calculates the patient's total blood volume (TBV) using Nadler´s equation. Post-collection Hct is then estimated by the system based on the TBV, volume of RBCs collected and amount of compensation fluid to be delivered. Divided in two equal collection cycles, 442 grams of RBCs were removed by each procedure. The plasma and buffy coat were returned to the patient after each cycle together with 400 mL of saline as a fluid compensation at the end.

Since 2017, therapeutic erythrocytapheresis has also been performed on the Spectra Optia system, using an automated Red Blood Cell Exchange procedure (RBCX). As the Spectra Optia system is a continuous-flow cell separator, it requires two veins for vascular access. The patient`s TBV is calculated using the Nadler`s method. The operator enters the desired patient Hct and the system calculates the required RBC volume to be depleted in order to reach the post-donation Hct target.

Both cell separators use the ACD-A anticoagulant solution (acid-citrate-dextrose formula) in an ACD-A to whole blood ratio of 1:10; saline was used for fluid compensation.

At the time of the introduction of erythrocytapheresis using the Spectra Optia system, validation of the procedure was performed in order to compare the technical features and performance of cytoreduction between the apheresis devices. The results of the procedures performed with 15 patients who underwent 21 erythrocytapheresis procedures on both cell separators were analysed.

### Statistical analysis methods

Patient characteristics were obtained from the hospital information system. All data regarding apheresis procedure details and any related complications were collected from the apheresis database. Median and range, or frequency and percentage were calculated for quantitative and qualitative variables, respectively. Differences between the erythrocytapheresis procedures of the two cell separators were assessed using Student's *t*-test with statistical significance being set at 0.05.

## Results

During the analysed period, 1731 procedures were performed in 125 patients, 1634 (94.4%) with the Haemonetics MCS+ system and 97 (5.6%) with the Spectra Optia system.

In 442 (25.5%) procedures performed in 56 patients using the Haemonetics MCS+ cell separator, the complete blood count (CBC) was determined immediately before and after the procedure, and cytoreduction efficiency was analysed. Patient CBC laboratory values and details of the erythrocytapheresis procedures are shown in Table 1. Hb and Hct values were significantly reduced after erythrocytapheresis, 18.69% for Hb and 18.73% for Hct levels, respectively (p-value of both <0.001).

**Table 1:** Procedural features and cytoreduction efficiency of 442 erythrocytapheresis performed on the Haemonetics MCS+ cell separator.

Parameter	Mean ± SD
Inlet volume (mL)	1017 ± 110
Anticoagulant volume (mL)	62 ± 10
Saline replacement volume (mL)	400 ± 0
RBCs removed (mL)	442 ± 0
Run time (min)	37 ± 5
Hb pre-procedure (g/L)	154.49 ± 15.20
Hct pre-procedure (%)	46.83 ± 4.74
Hb post-procedure (g/L)	125.67 ± 16.06
Hct post-procedure (%)	38.09 ± 5.49
Hb reduction (%)	18.69 ± 7.29
Hct reduction (%)	18.73 ± 8.54

SD: Standard deviation; RBCs: Red blood cells; Hct: Hematocrit; Hb: Hemoglobin.

During 442 erythrocytapheresis procedures performed using the Haemonetics MSC+ cell separator, only three (0.68%) vasovagal reactions (VVR) without loss of consciousness occurred and no procedure was interrupted due to these adverse reactions. Symptoms of VVR disappeared rapidly by reclining the patient into the Trendelenburg position. The erythrocytapheresis procedure was interrupted however in four (0.91%) cases: three procedures due to device technical failure, and one due to poor vascular access caused by vein rupture.

Fifteen patients were enrolled for a validation of the erythrocytapheresis procedure using the Spectra Optia system, and their demographics are shown in Table 2. The records of 21 procedures performed on the Spectra Optia separator were compared with the records of 21 previous treatments of the same patients on the Haemonetics MCS+ device, selected by comparable pre-apheresis values. Table 3 summarizes the comparisons of laboratory values of the 15 patients and features of the 42 erythrocytapheresis procedures on both cell separators. Median blood volumes of 991 mL (range: 815–1052 mL) and 1052 mL (range: 964–1267 mL) were processed during 18 min (range: 15–34 min) and 36 min (range: 31–45 min) on the Spectra Optia and Haemonetics MCS+ devices, respectively. For the Spectra Optia cell separator, the median reductions of Hb and Hct were 13.3% (range: 9.7–18.7%) and 14.3% (range: 10.16–20.0%), respectively. The median reductions of Hb and Hct for the Haemonetics MCS+ device were 21.5% (range: 13.9–25.2%) and 23.0% (range: 14.9–28.4%), respectively.

**Table 2:** Characteristics of 15 patients enrolled in a validation of erythrocytapheresis procedure on the Spectra Optia System.

Parameter	n or median (range)
Male / Female	13 / 2
Age (years)	53 (26–73)
Weight (kg)	92 (64–113)
Height (cm)	180 (158–190)
TBV (mL)	5600 (3705–6381)
Diagnosis	
Hemochromatosis	8
Polycythemia rubra vera	7

TBV: Total blood volume.

**Table 3:** Patient's laboratory values and details of the erythrocytapheresis procedures performed on the Spectra Optia and Haemonetics MCS+ separators.

	Spectra Optia (*n* = 21) median (range)	Haemonetics MCS+ (*n* = 21) median (range)	p-value
Hb before ECF (g/L)	147 (134–172)	150 (131–176)	0.676
Htc before ECF (%)	45 (40–48)	44.7 (39.3–50.8)	0.373
Hb after ECF (g/L)	129 (109–154)	119 (99–150)	<0.001
Htc after ECF (%)	39 (0.32–0.45)	34.2 (28.3–42.8)	<0.001
Hb reduction (%)	13.3 (9.7–18.7)	21.5 (13.9–25.2)	<0.001
Htc reduction (%)	14.3 (10.16–20.0)	23.0 (14.9–28.4)	<0.001
Blood volume processed (mL)	911 (815–1052)	1052 (964–1267)	<0.001
ACD-A (mL)	70 (63–81)	74 (68–84)	0.01
Saline replacement (mL)	429 (334–513)	400	0.209
Depleted RBC volume (mL)	495 (396–590)	442	<0.001
Procedure time (min)	18 (15–34)	36 (31–45)	<0.001

RBC: Red blood cell; ECF: Erythrocytapheresis Hct: Hematocrit; Hb: Hemoglobin; ACD-A: acid-citrate-dextrose anticoagulant.

The volume of saline replacement during apheresis (429 mL versus 400 mL for the Spectra Optia and Haemonetics MCS+ devices, respectively) did not differ significantly (p-value = 0.209) unlike the volume of citrate solution administered (70 mL versus 74 mL for Spectra Optia and Haemonetics MCS+, respectively; p-value = 0.01). The Spectra Optia procedure depleted a significantly higher RBC volume (495 mL versus 442 mL) in a shorter time (18 min versus 36 min).

No adverse reactions were recorded during the 21 procedures of the Spectra Optia device, while during the 21 procedures using the Haemonetics MCS+ cell separator, adverse reactions in the form of perioral tingling were recorded in three occasions. No erythrocytapheresis was interrupted due to medical reasons.

## Discussion

This study presents our long-term experience of erythrocytapheresis treatment for PV and HH, firstly evaluating cytoreduction by erythrocytapheresis in 442 patients treated using an intermittent-flow apheresis device, while the second part of the study presents a comparison of the erythrocytapheresis procedure details between intermittent- and continuous-flow cell separators.

Erythrocytapheresis as a method of cytoreduction is an efficient alternative to traditional therapeutic phlebotomy with the number of erythrocytapheresis procedures in patients with HH or PV having increased in recent years.^2^^,^^7^ An extensive survey conducted by Iona et al. on the practice in Italian transfusion centres showed great diversity in the practice, a need for clear guidance and a prior selection of patients who would benefit from this treatment to achieve the optimal cost-benefit balance.^8^

The history of erythrocytapheresis in our institution dates back to 2001 when the Haemonetics MCS+ cell separator was introduced for the collection of pre-operative autologous RBC units as well as for therapeutic procedures. Since then 1731 procedures have been performed on 125 patients as requested by attending physicians. The median duration of erythrocytapheresis therapy of five years with a median of ten procedures per patient show that it is a continuous and long-term treatment for conditions such as PV and HH.^5^ Numbers of patients with PV and HH are almost equal (42.4% and 47.2%, respectively). All these patients were previously treated with therapeutic phlebotomy, which is a simple and cheap method used for decades. Compared with erythrocytapheresis, phlebotomy has lower technical, medical and economical demands, but compliance to regular phlebotomy treatment differs. Rehaček et al. and Rombout-Sestrienkova et al. point out that one half and up to nearly two thirds of patients, respectively found regular phlebotomy uncomfortable and required reductions in their frequencies.^9^^,^^10^

In situations such as symptomatic PV or severe HH, when a rapid reduction of Hct or removal of serum iron is required, erythrocytapheresis is the therapy of choice. Currently, there is no standard protocol for RBC depletion. The use of erythrocytapheresis depends on the patient's diagnosis and the target Hct at the end of the procedure. In the studies published so far, different approaches were employed to achieve RBC depletion using automated methods. In the studies of two authors on erythrocytosis treatment, Evers et al. and Vecchio et al. targeted Hct levels below 45%.^11^^,^^12^ In the treatment of HH, Grabmer et al. presented a volume of reduced erythrocytes equivalent to the amount of removed serum iron in milligrams,^13^ and Rehacek determined the amount of removed erythrocytes in the range of 25–35%.^9^ Sundic et al. targeted a fixed volume of depletion (400 mL) as was the case in our erythrocytapheresis procedures using the Haemonetics MCS+ device.^14^ During the procedures using the Haemonetics MCS+ device, a fixed amount of 442 grams of concentrated RBC was depleted according to the settings of the protocol used. Due to the heterogeneity of diagnoses (HH and PRV) in the current study, the effect of 442 erythrocytapheresis procedures was shown as percentage reductions in Hb and Hct of 18.69% and 18.73%, respectively. As expected, there was a significant decrease (p-value <0.001) in post-apheresis values of Hct and Hb.

In this study, we compared the characteristics of erythrocytapheresis procedures between the Haemonetics MCS+ and Spectra Optia separators. Unlike the fixed volume of removed RBCs of the Haemonetics MCS+ separator, the RBC depletion procedure of the Spectra Optia device allows adjustments in the RBC volume according to the patient's characteristics and the target post-apheresis Hct level. In the present study, significantly more RBCs were removed employing the Spectra Optia device than the Haemonetics MCS+ device (*p*-value <0.001). The median time of the procedure utilising the Spectra Optia was significantly shorter in comparison to the Haemonetics MCS+ separator (18 min versus 36 min; p-value <0.001), making it more convenient for patients. Grabmer et al. describe an even shorter average duration of the procedure with the Spectra Optia separator (12.0 ± 0.4 min).^13^ Both separators are effective in cytoreduction, although there is a significant difference in the percentage of their reductions. Using the Haemonetics MCS+ separator the values of Hb and Hct were reduced by 21.5% and 23.0%, respectively (*p*-value <0.001), and for the Spectra Optia device they were 13.3% and 14.3% (*p*-value <0.001), respectively.

Regarding adverse reactions, the use of an apheresis system generally leads to a reduction of adverse events caused by volume depletion, most likely due to the use of saline compensation of removed RBC volume and longer blood collection times during apheresis.^16^, ^17^, ^18^ The frequency of apheresis-related adverse events in the literature varies from <2% up to 32.5%.^9^^,^^12^^,^^19^ In this study, during 442 erythrocytapheresis collections, only three (0.68%) VVR without loss of consciousness occurred. The pathophysiology of VVR is not completely understood but the presumed mechanism is related to the activity of peripheral baroreceptors of the donor. The donor's age, blood pressure and emotional state can affect these receptors.^20^ Likewise, the baroreceptor response is related to the percentage of donor blood volume removed.^20^^,^^21^ The low percentage of VVR in this study can be explained by careful selection of patients and the use of saline replacement during treatment. It is worth mentioning that the patient's emotional state can influence the occurrence of this adverse reaction, especially in first-time donors. In all our patients, when indicated for RBC depletion, standard therapeutic phlebotomy was performed first, and erythrocytapheresis was recommended only in patients who tolerated this procedure well. We included only hemodynamically stable patients, because volume shift in apheresis devices with intermittent flow has been considered challenging in patients with previous cardiovascular or neurological disease.

On comparing the procedures of the two devices, no adverse reactions were recorded on the Spectra Optia separator, while three reactions were recorded with the Haemonetics MCS+ device. All recorded adverse reactions refer to perioral tingling as a symptom of hypocalcemia, which was observed at the end of the cycles when plasma was being returned to the patient. Given that the volume of infused citrate did not differ significantly between these two devices (p-value = 0.01), the symptoms of hypocalcemia were probably caused not by total volume of infused citrate solution, but by a higher infusion rate at the end of the cycles.

The present study has some limitations. Firstly, due to its retrospective nature, serum ferritin values were not available in the medical records of all patients. A single erythrocytapheresis procedure significantly reduces more iron than a single phlebotomy, and generally achieves iron depletion in patients with HH in a shorter treatment period than phlebotomy.^10^ Secondly, an economic analysis of the cost of the erythrocytapheresis procedure, which is cited as a negative aspect of this therapeutic method, was not made in this setting, although it is well known that apheresis procedures have a higher price. As reported by Vecchio et al. and Rombout-Sestrienkova et al., erythrocytapheresis is 3.0- to 3.5-fold more expensive than phlebotomy. The higher costs can be explained by the higher price of the device or by indirect costs due to the longer time required by specialized apheresis staff. A longer interval between erythrocytapheresis procedures can only partially reduce the difference in total costs. However, among working-age HH patients, erythrocytapheresis results in less hours lost from work and a lower cost of lost production, with an overall cost per procedure one-third lower compared to phlebotomy.^10^^,^^12^^,^^15^

In conclusion, this study showed that erythrocytapheresis is a safe and effective method of cytoreduction. Although the cytoreductive effect is higher than in phlebotomy, the use of the erythrocytapheresis procedure is limited to patients with good venous access and without cardiovascular and neurological deficits. A comparison of two cell separators showed that the reduction of Hb and Hct was significantly higher in the Haemonetics MCS+ procedure. An additional advantage of the Haemonetics MCS+ cell separator is the venous access via a single vein, which most patients prefer. On the other hand, the main advantage of continuous-flow apheresis device Spectra Optia is the significantly shorter time of the procedure with normovolemia maintained throughout the procedure due to the continuous flow. Although the cost of erythrocytapheresis is higher compared to phlebotomy, more effective cytoreduction, which overall reduces the frequency of procedures and extends the interval between them, justifies the therapeutic use of erythrocytapheresis especially in medically eligible patients of working age. Availability of different apheresis devices allows for better tailoring of the procedures to specific patient needs.

## Conflicts of interest

The authors have disclosed no conflict of interest**.**
